# Heritability of variation in glycaemic response to metformin: a genome-wide complex trait analysis

**DOI:** 10.1016/S2213-8587(14)70050-6

**Published:** 2014-05-29

**Authors:** Kaixin Zhou, Louise Donnelly, Jian Yang, Miaoxin Li, Harshal Deshmukh, Natalie Van Zuydam, Emma Ahlqvist, The Wellcome Trust Case Control Consortium 2, Chris C Spencer, Leif Groop, Andrew D Morris, Helen M Colhoun, Pak C Sham, Mark I McCarthy, Colin N A Palmer, Ewan R Pearson

**Affiliations:** a Medical Research Institute, University of Dundee, Dundee, UK; b University of Queensland, Queensland Brain Institute, Brisbane, QLD, Australia; c Department of Psychiatry, Centre for Genomic Sciences and State Key Laboratory in Brain and Cognitive Sciences, LKS Faculty of Medicine, The University of Hong Kong, Hong Kong, China; d Oxford Centre for Diabetes, Endocrinology and Metabolism, University of Oxford, Oxford, UK; e Oxford NIHR Biomedical Research Centre, University of Oxford, Oxford, UK; f Churchill Hospital, and Wellcome Trust Centre for Human Genetics, University of Oxford, Oxford, UK; g Department of Clinical Sciences, Diabetes and Endocrinology, Lund University, University Hospital Skåne, Malmö, Sweden

## Abstract

**Background:**

Metformin is a first-line oral agent used in the treatment of type 2 diabetes, but glycaemic response to this drug is highly variable. Understanding the genetic contribution to metformin response might increase the possibility of personalising metformin treatment. We aimed to establish the heritability of glycaemic response to metformin using the genome-wide complex trait analysis (GCTA) method.

**Methods:**

In this GCTA study, we obtained data about HbA_1c_ concentrations before and during metformin treatment from patients in the Genetics of Diabetes Audit and Research in Tayside Scotland (GoDARTS) study, which includes a cohort of patients with type 2 diabetes and is linked to comprehensive clinical databases and genome-wide association study data. We applied the GCTA method to estimate heritability for four definitions of glycaemic response to metformin: absolute reduction in HbA_1c_; proportional reduction in HbA_1c_; adjusted reduction in HbA_1c_; and whether or not the target on-treatment HbA_1c_ of less than 7% (53 mmol/mol) was achieved, with adjustment for baseline HbA_1c_ and known clinical covariates. Chromosome-wise heritability estimation was used to obtain further information about the genetic architecture.

**Findings:**

5386 individuals were included in the final dataset, of whom 2085 had enough clinical data to define glycaemic response to metformin. The heritability of glycaemic response to metformin varied by response phenotype, with a heritability of 34% (95% CI 1–68; p=0·022) for the absolute reduction in HbA_1c_, adjusted for pretreatment HbA_1c_. Chromosome-wise heritability estimates suggest that the genetic contribution is probably from individual variants scattered across the genome, which each have a small to moderate effect, rather than from a few loci that each have a large effect.

**Interpretation:**

Glycaemic response to metformin is heritable, thus glycaemic response to metformin is, in part, intrinsic to individual biological variation. Further genetic analysis might enable us to make better predictions for stratified medicine and to unravel new mechanisms of metformin action.

**Funding:**

Wellcome Trust.

## Introduction

Metformin is the recommended first-line oral agent for the treatment of hyperglycaemia in patients with type 2 diabetes, with more than 100 million users worldwide. Despite its impressive safety record and efficacy at the population level, the exact mechanism of metformin action is still elusive and patients' glycaemic responses to metformin vary considerably.^1^, ^2^, ^3^ Understanding the source of such variation might help to identify patients most likely not to respond to metformin and could help to develop more effective agents by providing insight into the biological mechanism of metformin.

As with other complex traits, glycaemic response to metformin is probably determined by the interplay between genetic and environmental factors. Clinical variables such as BMI, drug adherence, and dosing only account for part of the variation.^3^ Pharmacogenetic studies have identified a few variants in genes affecting metformin action or its pharmacokinetics, yet these variants only account for a small fraction of the variation in metformin response.^4^, ^5^, ^6^, ^7^, ^8^ Two possible explanations have been suggested for why so little genetic contribution to metformin response variability has been identified. First, it might be because the overall genetic contribution to variation in glycaemic response to metformin is low, with variation mainly due to environmental factors; in this case, trying to improve understanding of the genetic and biological variation in metformin response would have little value. A second explanation is that variation in response to metformin does have a large genetic component, but so far most of the variants with small to moderate effects have not been identified in genetic association studies because of inadequate statistical power; in this case, effort and resources should be invested in an effort to discover the genetic contribution to metformin response because it might enable a truly stratified approach to treatment with this drug. Estimation of the extent of genetic contribution to glycaemic response to metformin—often termed heritability—is of key importance to understand which of these explanations is correct.

Historically, the heritability of drug response has rarely been established, largely because of the impracticality of applying the traditional twin and family study designs to drug-response phenotypes; assembling sufficient family members with the same diagnosis who have received the same medication and have been assessed using the same treatment outcome is all but impossible. Alternative methods using population-based genome-wide association study (GWAS) data for heritability estimation have been developed.^9^, ^10^, ^11^ One of these methods, genome-wide complex trait analysis (GCTA), can estimate the distant genetic relationship between unrelated individuals using GWAS single-nucleotide polymorphism (SNP) data and can correlate the genetic similarity to the phenotypic similarity, thus partitioning the total phenotypic variance into genetic and environmental causes. Since modern GWAS arrays have good coverage of most common variants in the human genome, the genetic variance estimated by the GCTA method—often referred to as chip heritability—is a good indicator of the additive genetic contribution from common SNPs.^12^ Because of the insufficient coverage of rare variants on GWAS arrays, heritability estimates by the GCTA method are often lower than the narrow-sense heritability derived from traditional twin and family studies. However, the GCTA method offers a more relevant and accurate estimate of drug-response heritability than other approaches that have been done using cell lines or animal models.^13^ In this study, we apply the GCTA method to GWAS data from the Genetics of Diabetes Audit and Research in Tayside Scotland (GoDARTS) study^14^ with the aim of establishing the heritability of glycaemic response to metformin.

## Methods

### Samples

In this GCTA study, we used a bioresource linked to electronic health record data (GoDARTS) rather than a specific cohort developed to assess metformin pharmacogenetics. As part of the Wellcome Trust UK type 2 diabetes case-control collection, patients with type 2 diabetes in Tayside, Scotland, have been invited to give written informed consent for DNA collection since October, 1997. So far, nearly 10 000 patients with type 2 diabetes have participated in the GoDARTS study.^14^ All clinical information about these patients can be obtained in an anonymised form from SCI-Diabetes (an electronic medical record for all patients with diabetes in Scotland) and these data are linked to biochemistry records and prescription encashments from 1992 onwards, giving a comprehensive longitudinal record of diabetes-related therapy. Participants consented for their data to be used in research into diabetes and related disorders, and this bioresource was approved by Tayside Regional Ethics Committee. The bioresource is now governed by Tayside Tissue Bank, which has approved the use of the bioresource for the study of metformin pharmacogenetics.

### Glycaemic response phenotypes

We used HbA_1c_ concentration, which is a routinely measured clinical test of glycaemic control in patients with diabetes, to establish glycaemic response to metformin (appendix); fasting glucose or other non-HbA_1c_ measurements of glycaemic control are not available in the GoDARTS study. Pretreatment (baseline) HbA_1c_ was defined as the measurement closest to, and within 6 months of, the metformin start date (index date), whereas on-treatment HbA_1c_ was defined as the minimum recorded HbA_1c_ achieved within 18 months after the index date. We used four different response phenotypes: absolute reduction in HbA_1c_, which was the difference between baseline and on-treatment HbA_1c_; proportional reduction in HbA_1c_, which was the absolute reduction divided by baseline HbA_1c_; adjusted reduction in HbA_1c_, which was the residuals of absolute reduction adjusted by known clinical covariates such as baseline HbA_1c_, adherence, dose, creatinine clearance, and treatment group; and a dichotomous phenotype of whether or not the target on-treatment HbA_1c_ of <7% (53 mmol/mol) was achieved, with adjustment for baseline HbA_1c_ and known clinical covariates.

Patients who received metformin monotherapy used no other antidiabetic drugs in the 6 months before the index date or during the study period; sulfonylurea treatment was continued throughout the study period in patients who used metformin as an add-on therapy. The sulfonylurea dose was allowed to vary. Details about how the covariates are defined, and the response models, are outlined in the appendix.

### GWAS data and quality control

GWAS data in the GoDARTS cohort were available from two previous studies. The Wellcome Trust Case Control Consortium 2 study (WTCCC2)^8^ genotyped 4134 patients with the Affymetrix 6.0 microarray (Santa Clara, CA, USA). The SUrrogate markers for Micro- and Macro-vascular hard endpoints for Innovative diabetes Tools (SUMMIT) study genotyped 3499 patients with the Illumina HumanOmniExpress microarray (Illumina, San Diego, CA, USA). Imputation to the HapMap3 panel—a database of common genetic variants that occur in human beings—and a stringent quality control pipeline were used to combine the two datasets and reduce the systematic discrepancy between the genotypes produced by the two microarrays and their corresponding calling algorithms (appendix).

We did two benchmark analyses with GCTA to validate the combined GWAS dataset. The first analysis showed that the heritability of human height was 46% (SE 6) in this cohort, which was consistent with previous estimates by studies applying the GCTA method.^15^ The second analysis estimated the heritability of a pseudo case-control phenotype assuming that samples from one genotyping platform were cases and those from the other platform were controls. As expected, the estimated heritability of this dummy platform phenotype was less than 1% (SE 5), confirming that the original GWAS datasets were combined without introduction of artificial heritability.

### Heritability estimation

We used GCTA version 1.11 to calculate the pair-wise genetic relationship between individuals and create the genetic relationship matrix.^13^ We then applied principal components analysis to all the SNPs to calculate the first ten eigenvectors, which we included as covariates in all the heritability estimation analyses to control for potential population structure. We then estimated univariate heritability of each drug-response phenotype by the restricted maximum likelihood method in GCTA, with sex and age at index date included as covariates.

Additionally, we used a bivariate analysis to jointly estimate the heritability of baseline HbA_1c_ concentrations and the heritability of on-treatment HbA_1c_ concentrations. The most informative parameter estimated from this bivariate analysis was the genetic correlation (*r*_g_), which represents the proportion of variance shared between baseline HbA_1c_ and on-treatment HbA_1c_ concentrations that was contributed by common genetic determinants. The correlation between the residual variance is *r*_e_, which represents, in part, contribution from environmental factors.

We established statistical significance using the likelihood-ratio test of specific hypothesis. We report the asymptotic 95% CI, which was calculated as 1·96 times the SE. Because the SEs of the parameter estimates were derived from first-order Taylor series expansions about the likelihood in GCTA, they might be biased for moderate study sample sizes,^15^ which at borderline levels of significance explains the discrepancy between p value and 95% CI reported.

### Role of the funding source

The sponsor had no role in study design, data collection, data analysis, data interpretation, or writing of the report. The corresponding authors had full access to all the data in the study and had final responsibility for the decision to submit for publication.

## Results

The combined dataset included 1 150 943 autosomal SNPs from 6992 patients. After filtering for cryptic relatedness, 5386 independent individuals were included in the final dataset. Of these, 2085 patients had sufficient clinical data to define their glycaemic response to metformin phenotypes. Table 1 summarises the main characteristics of the 2085 patients included in this study (for sample selection procedure see appendix), stratified by 1465 patients on metformin monotherapy and 620 patients who received metformin as add-on therapy to sulfonylureas.

**Table 1:** Sample characteristics

	**Metformin monotherapy (n=1465)**	**Metformin plus sulfonylureas (n=620)**
Age, years	61·4 (10·5)	65·4 (9·4)
Men	836 (57%)	390 (63%)
BMI, kg/m^2^	32·6 (5·6)	29·1 (4·9)
Baseline HbA_1c_, %	8·7 (1·3)	9·2 (1·3)
Baseline to metformin,* days	18 (29)	21 (30)
On-treatment HbA_1c_, %	7·0 (1·0)	7·4 (1·1)
Metformin dose, g/day	1·26 (0·47)	1·29 (0·51)
Adherence, %	78·4 (16·6)	78·3 (11·1)
Creatinine clearance, mL/min	96·1 (32·7)	79·5 (27·0)
HbA_1c_ measurements, n	3·9 (1·8)	4·2 (1·9)

Data are mean (SD) or number (%).

* Time from baseline measurement of HbA_1c_ to initiation of metformin treatment.

Heritability (*h*^2^) for baseline HbA_1c_ was 29% (95% CI −1 to 60; p=0·048 for the null hypothesis of being non-heritable), increasing to 42% (10–73; p=0·0052) for on-treatment HbA_1c_ (table 2). Baseline-adjusted on-treatment HbA_1c_ had a heritability of 36% (95% CI 4–69; p=0·011). Of the four drug-response phenotypes, the model-adjusted reduction in HbA_1c_ (*h*^2^=34%, 95% CI 1–68; p=0·022) and the ability to reach target HbA_1c_ (*h*^2^=32%, −1 to 64; p=0·030) were the most heritable. The heritability estimates for absolute reduction in HbA_1c_ (*h*^2^=23%, 95% CI −8 to 54) and proportional reduction in HbA_1c_ (*h*^2^=20%, 95% CI −11 to 51) were smaller, and were not statistically significant (table 2).

**Table 2:** Univariate heritability estimates of glycaemic response to metformin

	**n**	**Heritability (*h*^2^)**	**95% CI**	p value*
**HbA**_1c_**concentrations**				
Baseline HbA_1c_	2085	29%	−1 to 60	0·048
On-treatment HbA_1c_	2085	42%	10 to 73	0·0052
Adjusted on-treatment HbA_1c_	2085	36%	4 to 69	0·011
**Response phenotypes**				
Absolute reduction in HbA_1c_	2085	23%	−8 to 54	0·074
Proportional reduction in HbA_1c_	2085	20%	−11 to 51	0·10
Adjusted reduction in HbA_1c_	2069	34%	1 to 68	0·022
Achieved target HbA_1c_ concentration†	1942	32%	−1 to 64	0·030

* p values are from likelihood tests of null hypothesis of heritability being 0.

† The sample size was reduced to 1942 because some patients had a baseline HbA_1c_ concentration of 7% or lower.

To assess whether the genetic contribution to variation in response to metformin is driven by a few loci with a large effect or many loci with small effect, we did univariate heritability estimations for each chromosome separately for the two glycaemic response phenotypes that were significantly heritable. The genetic contribution to variation in response is distributed across several chromosomes (figure). When the proportion of variance in the model-adjusted reduction in HbA_1c_ attributable to each chromosome (chromosome-wise heritability) was regressed against the chromosome length, we noted a significant linear trend (p=0·037) for longer chromosomes to explain larger proportions of the variance. We also noted a similar trend (p=0·034) for achievement of target HbA_1c_.

**Figure:**
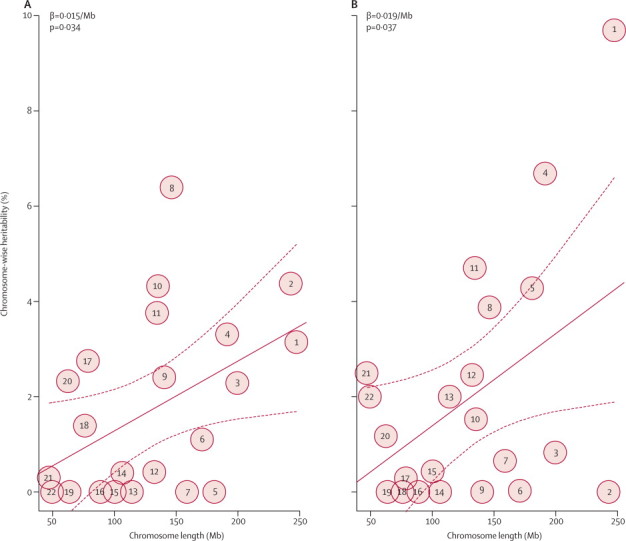
Chromosome-wise heritability estimation for glycaemic response to metformin Chromosome-wise heritability plotted for whether or not the target of on-treatment HbA_1c_<7% (53 mmol/mol) was achieved (A), and for model-adjusted reduction in HbA_1c_—ie, residuals of absolute reduction adjusted by known clinical covariates (B). The circled numbers show the heritability point estimates of each chromosome (sex chromosomes were not included). The solid lines plot the linear regression of chromosome-wise heritability against chromosome length; the dotted lines show 95% CI.

Bivariate analysis of baseline and on-treatment HbA_1c_ concentrations estimated a moderate genetic correlation (*r*_g_) of 0·58 (95% CI 0·06–1·09) between these two traits (table 3). Likelihood ratio tests showed that the genetic correlation was statistically greater than 0 (p=0·053) and marginally less than 1 (p=0·097); where 0 would mean no genetic correlation and 1 would represent 100% genetic correlation.

**Table 3:** Bivariate analysis of baseline and on-treatment HbA_1c_

	**Point estimate**	**95% CI**
Baseline HbA_1c_	0·29	−0·02 to 0·60
On-treatment HbA_1c_	0·42	0·11 to 0·73
*r*_g_	0·58	0·06 to 1·09
*r*_e_	0·28	−0·02 to 0·58

The point estimates of baseline and on-treatment HbA_1c_ are for heritability; correlation for *r*_g_ (genetic) and *r*_e_ (environmental).

## Discussion

This study is, to our knowledge, the first to show that genetic differences contribute considerably to the variation noted in patients' glycaemic response to metformin (panel). The heritability estimates for the frequently used definitions of glycaemic response range from 20% to 34%, suggesting that genetic variants are likely to have an important contribution to variation in glycaemic response to metformin in patients with type 2 diabetes. In the context of GCTA estimates for other complex traits with well established heritability by family or twin studies, the point estimates are similar to GCTA estimates for schizophrenia (*h*^2^=23% [SE 1]) and Alzheimer's disease (*h*^2^=30% [SE 3]),^16^, ^17^ suggesting that genetic variants contribute to the variation in HbA_1c_ response to metformin to a similar extent.

Panel **Research in context**

**Systematic review**

We searched PubMed on Feb 18, 2013, with the search terms “heritability” and “metformin”. We did not apply any publication date or language restrictions. We found no previous reports on the heritability of glycaemic response to metformin.

**Interpretation**

This study is, to our knowledge, the first to establish that glycaemic response to metformin is likely to be moderately heritable. Enhanced GWAS studies will identify more variants, enabling better response predictions to be made, and will identify new mechanisms of metformin action in the reduction of hyperglycaemia in the treatment of type 2 diabetes.

We did the chromosome-wise heritability estimation to provide information about the genetic architecture of glycaemic response to metformin. Clearly, several variants across different chromosomes contribute to the metformin response variation. The finding that the contribution by an individual chromosome is significantly correlated to its length suggests that on each chromosome might be many variants with a small to moderate effect size rather than a few variants with major effect. This hypothesis is also supported by results of the metformin response GWAS, which reported that no individual variant explained a large proportion of the variance.^8^ Notably, the point estimates of chromosome-wise heritability all have large 95% CIs and the estimates for each chromosome vary between the two different response phenotypes (figure). Thus, individual extreme values, such as the estimate of chromosome 1 in the analysis of model-adjusted reduction in HbA_1c_, could have had an undue effect on the reported trend.

In the univariate GCTA analysis we were able to assess whether different metformin response phenotypes are heritable. We do not have statistical power to conclude that one phenotype is more heritable than another, although the point estimates for the heritability of the response phenotypes that adjusted for the baseline HbA_1c_ were greater than for the unadjusted models. Because baseline HbA_1c_ has been well documented to have a major effect on the absolute reduction phenotype,^18^ the higher heritability estimates for baseline-adjusted phenotypes of metformin efficacy are likely to be a result of the successful adjustment for common environmental variance between baseline and on-treatment HbA_1c_ measurements. These adjusted phenotypes probably best address the pharmacogenetics of metformin when considering what factors are associated with the greatest reduction in HbA_1c_ for a given HbA_1c_ concentration before metformin initiation. An adjusted phenotype was used to successfully identify variants near the *ATM* locus that affect on-treatment HbA_1c_ but not baseline HbA_1c_.^8^ However, such definitions adjusted for baseline HbA_1c_ do capture some of the shared genetic component (*r*_g_) described above, and thus identified variants might reflect not only the response to metformin, but also the variance in HbA_1c_ per se. When considering what the biological determinants of response to metformin are, a better phenotype might be the unadjusted absolute reduction in HbA_1c_ because this measure does not capture any shared genetic contribution, only the variants with differential genetic effects between the HbA_1c_ before and after initiation of metformin treatment.^19^ Such an HbA_1c_ reduction model unadjusted for the baseline measure has been used in studies of statin pharmacogenetics.^20^, ^21^ However, the heritability for the absolute reduction in HbA_1c_ did not achieve statistical significance in our study of response to metformin treatment.

We report a new application of the GCTA bivariate analysis in this drug-response study. This approach has advantages over univariate approaches because it does not make assumptions about the response model; rather, it uses a quantitative genetic approach to partition variance into genetic and environmental fractions that are shared and non-shared between two states or traits. The idea behind such an analysis is that intervention with metformin can change the physiological state of a patient. In the pretreatment state, a set of genetic and environmental factors determine the HbA_1c_ variation; a potentially different set of genetic and environmental determinants affect the on-treatment state (appendix). The bivariate analysis can tell us not only how much of the HbA_1c_ variance is genetically determined in each state, but also how much of the genetically determined HbA_1c_ variance is shared between the two physiological states, as estimated by genetic correlation (*r*_g_). The shared variants that underlie the genetic correlation have the same effect on HbA_1c_ variation in the two states, and their genetic contribution to HbA_1c_ is not changed by metformin treatment. Thus an *r*_g_ of 1 would imply that metformin intervention does not change the genetic determinants of HbA_1c_ in the pretreatment and on-treatment state—ie, no pharmacogenetic effect occurs.^20^ By contrast, a low *r*_g_ would imply that the genetic determinants of HbA_1c_ are largely different before and after metformin treatment, hence a strong pharmacogenetic effect. In our analysis, the point estimate for the shared genetic contribution was 0·58, suggesting that around half of the genetic determinants contributing to baseline HbA_1c_ and on-treatment HbA_1c_ concentrations were shared, with half the genetic determinants differing between the baseline and metformin treatment states; however, we do acknowledge that the 95% CI precludes a definitive conclusion of this bivariate analysis.

A key limitation of this study is the reasonably small sample size. However, the GoDARTS GWAS data used are from the largest metformin pharmacogenomic cohort done so far, including 2085 individuals who received metformin. Yet we still noted considerable 95% CIs for the heritability and genetic correlation estimates due to the limited sample size. Thus, despite having sufficient power to find that glycaemic response to metformin is a heritable trait, we do not have power to establish whether one drug response trait is more heritable than another. To do this, 4450 patients would be needed to statistically differentiate true heritability of 20% and 34%, which correspond to the two extremes of the estimated heritability of our four reported phenotypes.^22^ This shortfall in available data emphasises the importance of a consortium effort to assemble even more GWAS data, which will enable us to not only achieve more accurate estimates of heritability, but also discover more genetic variants that account for this heritability. The Metformin Genetics Consortium (MetGen) consists of research groups in Europe and the USA that have cohorts available for the study of the genetics of metformin. This consortium currently consists of about 5600 patients who have received metformin, and hopefully in the next 2–3 years additional academic and commercial clinical trial data and observational data might enable a GWAS of about 8000 individuals. Of note, interpretations of the heritability estimates from our current GCTA analyses can only be made in the context of the SNPs captured by the GWAS arrays. Contributions from the rare variants that are poorly covered by the GWAS panels will not form part of the heritability estimated by GCTA, but will remain in the environmental component. The observed environmental (residual) correlation (*r*_e_) of 0·28 could be contributed by both environmental factors and shared rare genetic variants. Thus sequencing-based genomic studies with an emphasis on the rare drug-response variants are valid irrespective of the heritability estimates from GWAS SNPs.

In summary, using GWAS data from 2085 patients with type 2 diabetes, our analysis showed that genetic variants contributed to the variation in glycaemic response to metformin, with the heritability of metformin response estimated at up to 34% (95% CI 1–68; p=0·022). This result shows that a moderate proportion of the variance in glycaemic response is genetic, and represents underlying biological differences between individuals. The variants are likely to have a small-to-moderate effect and be scattered across the genome. So far, very little of the genetic contribution to metformin response has been identified; GWAS analyses with larger samples could find more genetic variants that enable better predictions to be made for personalised or stratified medicine, and unravel new mechanisms of metformin action in the reduction of hyperglycaemia.
