# Supplementary material for: Heritability of variation in glycaemic response to metformin: a genome-wide complex trait analysis
Source: Lancet Diabetes Endocrinol. 2014 May 29;2(6):481–7. doi: 10.1016/S2213-8587(14)70050-6 (PMC4038749; doi:10.1016/S2213-8587(14)70050-6)
Supplement: Supplementary appendix [file mmc1.pdf]

THE LANCET Diabetes & Endocrinology

Supplementary appendix

This appendix formed part of the original submission and has been peer reviewed. We post it as supplied by the authors.

Supplement to: Zhou K, Donnelly L, Yang J, et al. Heritability of variation in glycaemic response to metformin: a genome-wide complex trait analysis. *Lancet Diabetes Endocrinol* 2014; published online March 19. http://dx.doi.org/10.1016/S2213-8587(14)70050-6.

Appendix

Appendix method 1. Defining metformin glycaemic response models in GoDARTS

Because over 92% of the OHA prescriptions issued in GoDARTS cohort are either metformin (51.4%) or sulphonylurea (41.2%), we focused on two treatment schemes of metformin monotherapy (metformin added in following failure of dietary control) or dual therapy (metformin added to stable sulphonylurea treatment).

Following initiation of oral hypoglycaemic agents in type 2 diabetes, there is an initial reduction in HbA1c, followed by a gradual deterioration. This can be seen in both the UKPDS study and other diabetes trials such as ADOPT. The gradual deterioration in HbA1c will reflect both drug efficacy (or inefficacy) to control HbA1c, and the underlying diabetes progression. To target the drug response alone we focused on the first 18 months of metformin therapy, to minimize the response window but ensure minimal exclusion due to lack of HbA1c data.

In this observational study, we used two HbA1c measures to define four metformin glycaemic response phenotypes that are commonly used in published metformin pharmacogenetic studies. The baseline HbA1c value used was the one closest to, but within -6 months and +7 days of index date. The on-treatment HbA1c was defined as the minimum HbA1c achieved between 1 and 18 months after metformin treatment or prior to a change in therapy (cessation of metformin or addition of further oral hypoglycaemic therapy).

Three types of quantitative traits that are commonly used in published metformin pharmacogenetic studies were investigated here. The absolute HbA1c reduction is the basic phenotype that places even weight on the variance in baseline and on-treatment HbA1c. The proportional reduction and multiple linear model (with baseline as a covariate) adjusted reduction are two different means of evaluating the metformin glycaemic response by controlling for the well established influence of baseline HbA1c on treatment efficacy. In this observational study, the patient's physician will be treating to achieve an HbA1c target, which over the majority of the study period would have been 7%. We therefore defined our dichotomous trait of metformin response phenotype as the ability to achieve a minimum HbA1c below 7%.

We used multiple linear or logistic regressions to explore the contribution of clinical covariates that contributed to drug response variance in this observational data set. The definitions of covariates were described below. Age, sex and weight were used to derive the creatinine clearance so were not included separately in the models. Although duration of diabetes has been well established as a strong predictor of treatment efficacy, it was not included due to unacceptable level of missingness. Appendix Table 1 describes the multivariate linear model of absolute HbA1c reduction and Appendix Table 2 describes the multivariate logistic regression model of the dichotomous trait of achieving a treatment target. Although treatment daily dose is a strong predictor of response, as indicated by the univariate R^2, it was not significant in the model due to collinearity with baseline HbA1c.

Appendix Method 2. Covariates Definitions

- ***Drug Adherence***: Adherence was estimated as:

  Adherence = sum (days covered by each prescription)/ days in the study period

  in which the days covered by a prescription was calculated as dividing the dispensing quantity by daily dose; if one prescription covered a time period beyond next prescription start, the extra days were not taken over to the calculation for next prescription.

- ***Drug Daily Dose***: The average daily dose during the 3 months prior to the minimum HbA1C was achieved
- ***Creatinine Clearance***: The creatinine clearance rate was calculated using the Cockcroft-Gault equation:

  *GFR = (140-age) * (weight in kg) * (0.85 if female) / (72 * creatinine in mg/dL)*

- ***Baseline Gap:*** The number of days between baseline and index date was used to account for the unobserved T2D progression during the gap
- ***Number of HbA1c Measurements:*** The number of HbA1c measurements recorded during the study period, reflecting the opportunity of being able to detect the real minimum HbA1c

Appendix Table 1. Multiple linear model of absolute HbA1c reduction

	**Beta (95% CI)**	**p-value**	**R^2**
Baseline HbA1c (%)	0.72 (0.69,0.75)	<0.0001	0.535
Baseline Gap (30 days)	-0.07 (-0.11,-0.03)	0.001	0.029
Average Dose Metformin (g/day)	0.14 (-0.22,0.51)	0.44	0.042
Adherence (%)	0.71 (0.47,0.95)	<0.0001	0.012
Creatinine Clearance (ml/10min)	-0.03 (-0.04,-0.01)	<0.0001	0.002
Number of HbA1c Measurements	0.12 (0.10,0.14)	<0.0001	0.033
Treatment Group (Dual-therapy group as reference)	0.31 (0.21,0.40)	<0.0001	0.003

The R^2 column for each covariate is from univariate analysis. The multiple R^2 is 0.58 for the above model. The residuals from this model was used as the phenotype for model adjusted reduction heritability analysis.

Appendix Table 2. Multiple linear model of proportional HbA1c reduction

	**Beta (95% CI)**	**p-value**	**R^2**
Baseline HbA1c (%)	5.61(5.28,5.93)	<0.0001	0.377
Baseline Gap (30 days)	-1.21(-1.67,-0.76)	0.001	0.038
Average Dose Metformin (g/day)	1.76(-2.06,5.65)	0.37	0.032
Adherence (%)	8.07(5.44,10.69)	<0.0001	0.018
Creatinine Clearance (ml/10min)	-0.34(-0.48,-0.20)	<0.0001	0.006
Number of HbA1c Measurements	1.32(1.07,1.56)	<0.0001	0.043
Treatment Group (Dual-therapy group as reference)	2.74(1.71,3.76)	<0.0001	0.002

The dependent variable is measured in percentage. The R^2 column for each covariate is from univariate analysis. The multiple R^2 is 0.443 for the above model.

Appendix Table 3. Multiple logistic regression model of achieving a target

	**OR (95% CI)**	**p-value**
Baseline HbA1c (%)	1.43(1.32,1.56)	<0.0001
Baseline Gap (30 days)	1.21(1.08,1.35)	0.0007
Average Dose Metformin (g/day)	1.18(0.98,1.47)	0.12
Adherence (%)	0.88(0.83,0.93)	<0.0001
Creatinine Clearance (ml/10min)	1.07(1.04,1.11)	<0.0001
Number of HbA1c Measurements	0.83(0.78,0.87)	<0.0001
Treatment Group (Dual-therapy group as reference)	0.49(0.39,0.62)	<0.0001

The cases are non-responders.

Appendix Figure 1. Sample ascertainment flow chart.

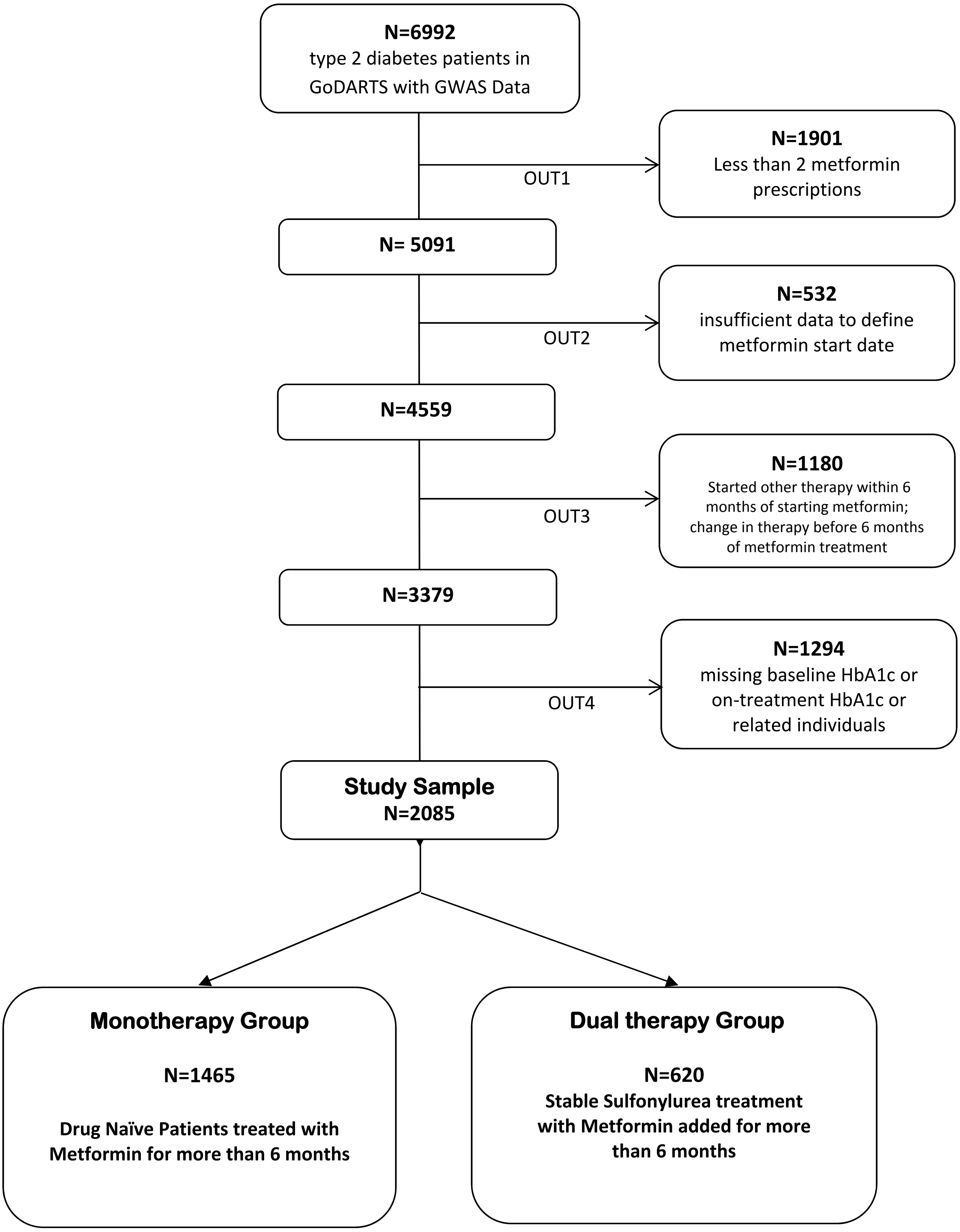

Appendix Figure 2. Genotyping quality control and imputation pipeline

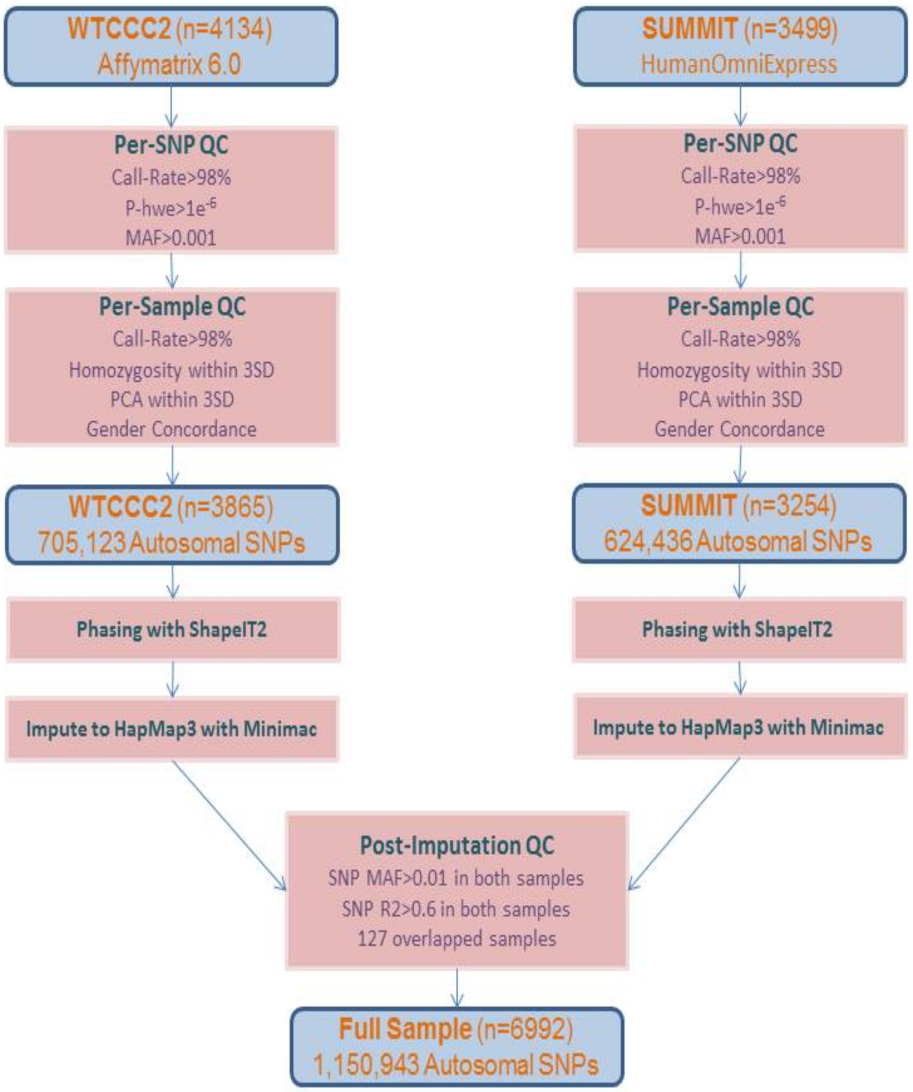

Appendix Figure 3. GCTA bivariate partitioning of variance in glycaemic response to metformin. In the pre-treatment state, the variance is partitioned into a genetic component of A_{pre} (additive genetic contribution from all GWAS SNPs) and an environmental component E_{pre} (all the residual variance not explained by GWAS SNPs). Similarly, in on-treatment state the genetic and environmental variance components are A_{on} and E_{on} respectively. r_g is the shared additive genetic variance across the two states as contributed by GWAS SNPs, and r_e is the correlation of the residual variance.

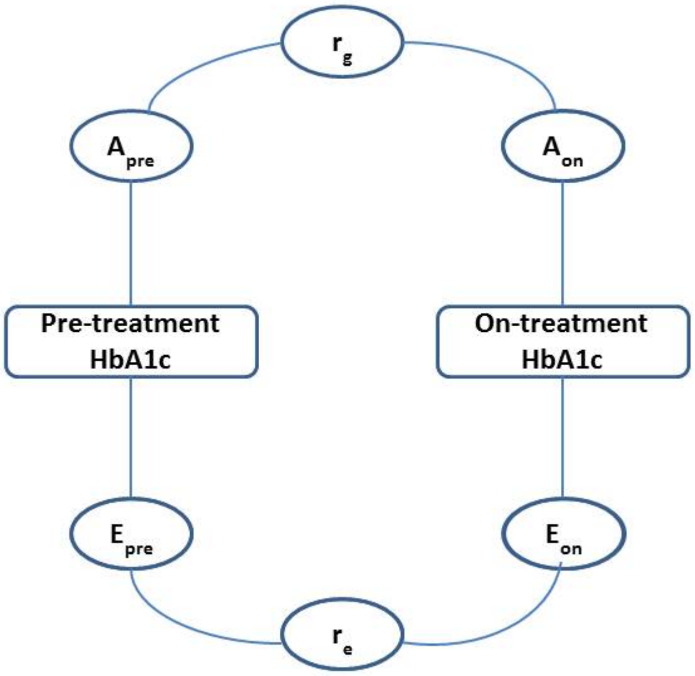

Appendix: Membership of Wellcome Trust Case Control Consortium 2

Management Committee

Peter Donnelly (Chair)[1,2], Ines Barroso (Deputy Chair)[3], Jenefer M Blackwell[4, 5], Elvira Bramon[6] , Matthew A Brown[7] , Juan P Casas[8] , Aiden Corvin[9], Panos Deloukas[3], Audrey Duncanson[10], Janusz Jankowski[11], Hugh S Markus[12], Christopher G Mathew[13], Colin NA Palmer[14], Robert Plomin[15], Anna Rautanen[1], Stephen J Sawcer[16], Richard C Trembath[13], Ananth C Viswanathan[17], Nicholas W Wood[18]

Data and Analysis Group

Chris C A Spencer[1], Gavin Band[1], Céline Bellenguez[1], Colin Freeman[1], Garrett Hellenthal[1], Eleni Giannoulatou[1], Matti Pirinen[1], Richard Pearson[1], Amy Strange[1], Zhan Su[1], Damjan Vukcevic[1], Peter Donnelly[1,2]

DNA, Genotyping, Data QC and Informatics Group

Cordelia Langford[3], Sarah E Hunt[3], Sarah Edkins[3], Rhian Gwilliam[3], Hannah Blackburn[3], Suzannah J Bumpstead[3], Serge Dronov[3], Matthew Gillman[3], Emma Gray[3], Naomi Hammond[3], Alagurevathi Jayakumar[3], Owen T McCann[3], Jennifer Liddle[3], Simon C Potter[3], Radhi Ravindrarajah[3], Michelle Ricketts[3], Matthew Waller[3], Paul Weston[3], Sara Widaa[3], Pamela Whittaker[3], Ines Barroso[3], Panos Deloukas[3].

Publications Committee

Christopher G Mathew (Chair)[13], Jenefer M Blackwell[4,5], Matthew A Brown[7], Aiden Corvin[9], Mark I McCarthy[19], Chris C A Spencer[1]

1 Wellcome Trust Centre for Human Genetics, Roosevelt Drive, Oxford OX3 7BN, UK; 2 Dept Statistics, University of Oxford, Oxford OX1 3TG, UK; 3 Wellcome Trust Sanger Institute, Wellcome Trust Genome Campus, Hinxton, Cambridge CB10 1SA, UK; 4 Telethon Institute for Child Health Research, Centre for Child Health Research, University of Western Australia, 100 Roberts Road, Subiaco, Western Australia 6008; 5 Cambridge Institute for Medical Research, University of Cambridge School of Clinical Medicine, Cambridge CB2 0XY, UK; 6 Department of Psychosis Studies, NIHR Biomedical Research Centre for Mental Health at the Institute of Psychiatry, King’s College London and The South London and Maudsley NHS Foundation Trust, Denmark Hill, London SE5 8AF, UK; 7 Diamantina Institute of Cancer, Immunology and Metabolic Medicine, Princess Alexandra Hospital, University of Queensland, Brisbane, Queensland, Australia; 8 Dept Epidemiology and Population Health, London School of Hygiene and Tropical Medicine, London WC1E 7HT and Dept Epidemiology and Public Health, University College London WC1E 6BT, UK; 9 Neuropsychiatric Genetics Research Group, Institute of Molecular Medicine, Trinity College Dublin, Dublin 2, Eire; 10 Molecular and Physiological Sciences, The Wellcome Trust, London NW1 2BE; 11 Centre for Digestive Diseases, Queen Mary University of London, London E1 2AD, UK and Digestive Diseases Centre, Leicester Royal Infirmary, Leicester LE7 7HH, UK and Department of Clinical Pharmacology, Old Road Campus, University of Oxford, Oxford OX3 7DQ, UK; 12 Clinical Neurosciences, St George's University of London, London SW17 0RE; 13 King’s College London Dept Medical and Molecular Genetics, School of Medicine, Guy’s Hospital, London SE1 9RT, UK; 14 Biomedical Research Centre, Ninewells Hospital and Medical School, Dundee DD1 9SY, UK; 15 King’s College London Social, Genetic and Developmental Psychiatry Centre, Institute of Psychiatry, Denmark Hill, London SE5 8AF, UK; 16 University of Cambridge Dept Clinical Neurosciences, Addenbrooke’s Hospital, Cambridge CB2 0QQ, UK; 17 NIHR Biomedical Research Centre for Ophthalmology, Moorfields Eye Hospital NHS Foundation Trust and UCL Institute of Ophthalmology, London EC1V 2PD, UK; 18 Dept Molecular Neuroscience, Institute of Neurology, Queen Square, London WC1N 3BG, UK; 19 Oxford Centre for Diabetes, Endocrinology and Metabolism (ICDEM), Churchill Hospital, Oxford OX3 7LJ, UK.
